# Segmental analysis of aortic basal ring dimensions in normal and dilated tricuspid aortic roots

**DOI:** 10.1093/icvts/ivae029

**Published:** 2024-02-28

**Authors:** Matija Jelenc, Blaž Jelenc, Sara Habjan, Christian Giebels, Peter Fries, Hector I Michelena, Thomas Foley, Hans Joachim Schäfers

**Affiliations:** Department for Cardiovascular Surgery, University Medical Center Ljubljana, Ljubljana, Slovenia; Faculty of Mathematics and Physics, University of Ljubljana, Ljubljana, Slovenia; Department for Cardiovascular Surgery, University Medical Center Ljubljana, Ljubljana, Slovenia; Department of Thoracic and Cardiovascular Surgery, Saarland University Medical Centre, Homburg, Germany; Clinic for Diagnostic and Interventional Radiology, Saarland University Medical Centre, Homburg, Germany; Department of Cardiovascular Medicine, Mayo Clinic, Rochester, MN, USA; Department of Radiology, Mayo Clinic, Rochester, MN, USA; Department of Thoracic and Cardiovascular Surgery, Saarland University Medical Centre, Homburg, Germany

**Keywords:** Aortic root dilatation, Aortic valve anatomy, Computed tomography angiography, Tricuspid aortic valve, Propensity score matching, Aortic valve annulus

## Abstract

**OBJECTIVES:**

In patients with aortic root aneurysm, the aortic basal ring is frequently dilated. It has been speculated that the muscular part of the basal ring dilates most. The purpose of this study was to analyse the segmental dilatation of the basal ring, comparing normal and dilated roots in patients with tricuspid aortic valves.

**METHODS:**

Retrospective analysis of computed tomography studies in patients with normal and dilated aortic roots was performed. Lengths of segments of the basal ring corresponding to each of the 3 sinuses, and to the muscular and fibrous parts were measured. Fractions of these segments relative to the total basal ring perimeter were calculated.

**RESULTS:**

We analysed 152 normal and 126 dilated aortic roots and 86 propensity-matched pairs. Basal ring dilatation was present in all segments of dilated aortic roots with subtle differences between the segments corresponding to the 3 sinuses. The muscular part of the basal ring dilated proportionately to its fibrous part, with no difference in fractions of measured muscular part in normal and dilated roots [42.2% (interquartile range 4.3%) vs 42.1% (interquartile range 6.3%)].

**CONCLUSIONS:**

Basal ring dilatation was present in all segments corresponding to the 3 sinuses in dilated aortic roots. Both muscular and fibrous parts dilated equally, supporting the need to stabilize the entire basal ring when performing aortic valve repair surgery.

## Graphical Abstract

**Figure:**
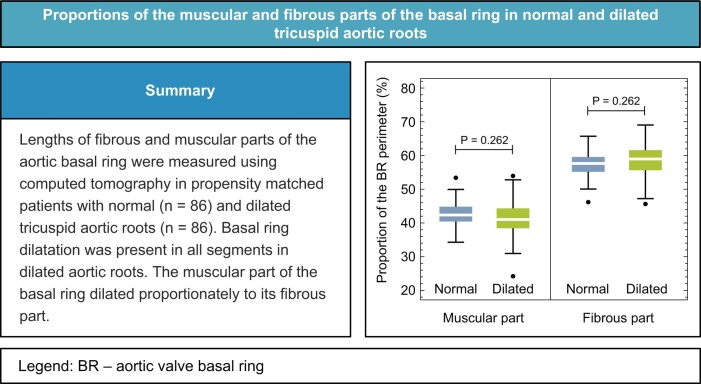

## INTRODUCTION

In patients with a dilated aortic root with or without concomitant aortic valve regurgitation, the basal ring (BR) representing the virtual perimeter at the level of nadirs of the 3 aortic sinuses, is frequently also dilated. The BR can be divided into muscular and fibrous parts. The muscular part includes the interventricular septum beneath the right coronary cusp and right–left commissure and left ventricular myocardium beneath the left coronary sinus. The fibrous part includes the left fibrous trigone located beneath the left coronary sinus, the aorto-mitral curtain and the right fibrous trigone with membranous septum beneath the right-non-coronary commissure [1, 2].

There is an ongoing debate regarding which part of the BR dilates most in patients with aortic root aneurysm. The fibrous part of the BR has long been thought to be primarily involved in dilatation [3]. However, in analogy to the mitral valve, where the posterior and muscular annulus is primarily dilated, the aortic root has recently been thought to dilate primarily in its muscular portion [4]. This could be an explanation for the observation that in the aortic valve, the right coronary cusp is most commonly affected by prolapse [5].

The aim of this study was to analyse the segmental dilatation of the BR comparing normal and dilated roots in tricuspid aortic valves. Using computed tomography angiography (CTA), we analysed the dimensions of the segments of the BR that correspond to each of the 3 sinuses. We also determined the relative proportions of muscular and fibrous parts of the BR and estimated the intertrigonal distance.

## PATIENTS AND METHODS

We retrospectively analysed ECG-synchronized CTA studies obtained from the 3 contributing centres picture archiving and communication systems. The CTAs were performed between March 2012 and May 2023. CTA datasets were anonymized; however, they included gender, age, height and weight of the patient in the metadata.

### Ethics

The study was approved by the local or national medical ethics committees (Komisija Republike Slovenije za medicinsko etiko, No. 0120-133/2021/3 and No. 0120-312/2022/3, Ärztekammer des Saarlandes No. 174/22, Mayo Clinic Institutional Review Board No. 14-005324). Written patient informed consent was waived as this was a non-interventional study with retrospective data acquisition and analysis.

### Inclusion and exclusion criteria

Normal roots were defined as the diameter of sinuses of Valsalva of <45 mm, dilated roots as equal or more than 45 mm [6]. For the normal root group, we used coronary CTAs of patients, where imaging was performed to rule out coronary disease or anomalies. Exclusion criteria were bicuspid aortic valve, poor contrast, poor aortic cusp visibility, motion artefacts, calcifications of the aortic valve cusps and aortic root or ascending aorta diameter equal or larger than 45 mm.

For the dilated root group, we used thoracic aorta CTAs or coronary CTAs of patients with aortic root and/or ascending aorta dilatation, who were diagnosed or treated in either of the 3 contributing centres due to dilated aorta and/or aortic regurgitation. Exclusion criteria were isolated dilatation of the ascending aorta with normal aortic root, bicuspid aortic valve, poor contrast, motion artefacts and poor cusp visibility.

### Valve analysis

**Figure 1:**
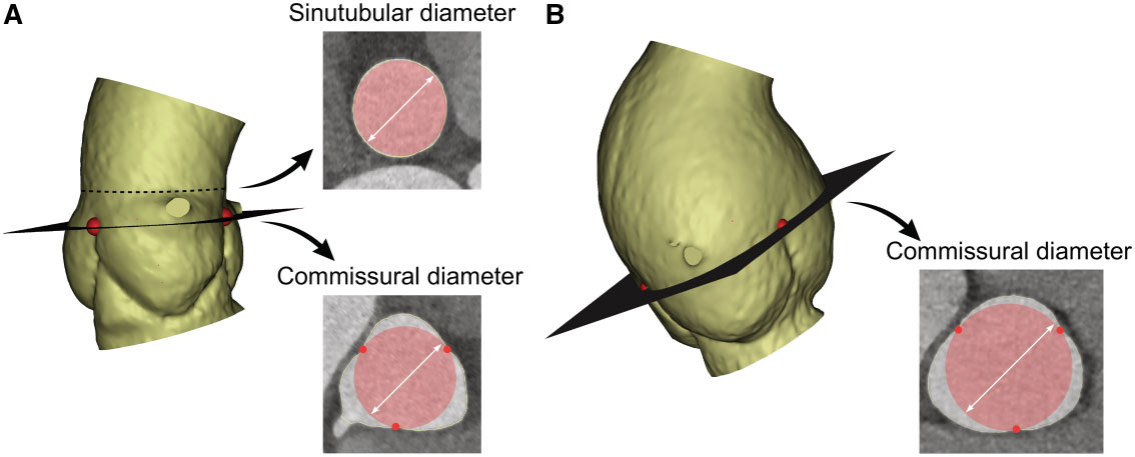
Definition of commissural diameter. (**A**) Normal aortic root has clearly visible sinutubular junction (dashed line). Showing cross-sections at the level of the sinutubular junction and the commissures. Commissural diameter was defined as the diameter of the virtual circle that would fit through the 3 commissures (shown in red). (**B**) Dilated aortic root with commissural cross-section. The sinutubular junction is absent.

All valve analyses were performed in the end-diastolic phase of the cardiac cycle. The CTAs were imported into Mimics Innovation Suite v. 21.0 (Materialise, Leuven, Belgium) where aortic roots were segmented. On each CTA, we marked the nadirs of the 3 sinuses (nadir of the right aortic sinus, nadir of the left aortic sinus, nadir of the non-coronary aortic sinus) and the 3 commissures [right–left commissure, left-non commissure, right-non commissure (RNC)]. The perimeter of the BR was then traced at the plane defined by the nadirs of the 3 sinuses with a closed spline tool. The open spline tool was used to trace the 3 cusp insertions. Diameter of sinuses of Valsalva was measured using maximal internal distance between the farthest points in the right and the non-coronary sinuses of Valsalva (‘sinus-to-sinus’) in a plane parallel to the BR plane [7]. The points and splines defined by three-dimensional (3D) coordinates were then imported into Mathematica v12.0 (Wolfram Research, UK) where dedicated code was used to reconstruct the aortic valve in 3D space from the measured points and splines and calculate different lengths for each patient. The BR spline was the basis for measurements of BR perimeter, maximal and minimal BR diameter. Maximal and minimal diameters were defined as the longest and the shortest diameters passing through the centroid of the BR, respectively. Mean diameter was calculated as perimeter divided by pi. The degree of ellipticity of the BR was estimated by calculating the ratio between maximal and minimal BR diameter. The sinutubular junction was clearly visible in normal aortic roots but was usually absent in dilated roots. To be able to compare the 2 groups, we used commissural diameter, which was defined as the diameter of a virtual circle that would fit through the 3 commissures (Fig. 1). Aortic valve cusps were qualitatively inspected on the CTAs for cusp prolapse, which was defined as a break in the radial contour of the cusp with lower effective height than the 2 opposing cusps.

### Segments of the basal ring perimeter corresponding to each of the 3 sinuses

**Figure 2:**
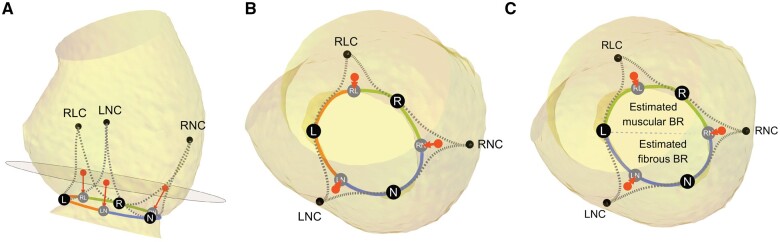
Segments of the basal ring. (**A**) Side view of the aortic root. RLC, LNC and RNC are right–left, left-non and right-non commissures, respectively. Red points mark the midpoints of inter-leaflet triangles at the level of the plane, which is one-third of the distance between basal ring plane and commissural plane. Red arrows point to projections of midpoints onto the basal ring (grey points RL, LN and RN). Black points on the basal ring mark the nadirs of the 3 sinuses (points L, R and N). (**B**) Top view of the aortic root. Segments of the basal ring corresponding to the 3 sinuses are marked with different colours (left—orange, right—green, non-coronary—blue). (**C**) Estimated muscular and fibrous basal ring. Top view of the aortic root, showing the basal ring split into 2 parts. The estimated muscular BR (green), which includes the right segment of the basal ring and a part of the adjacent left segment to the nadir of the left sinus. The estimated fibrous BR (blue), which includes the non-coronary segment of the basal ring and part of the adjacent left segment to the nadir of the left sinus.

An additional 3D plane was defined at one-third of the distance between BR plane and commissural plane starting from the BR plane, with its normal plane vector weighted as two-thirds of the BR plane normal vector and one-third of the commissural plane normal vector. At the level of this plane, intersections with cusp insertions were defined and for each inter-leaflet triangle, a midpoint at this plane was calculated as the mean of 2 opposing intersections. The 3 midpoints were then projected to the BR and were used as a border between segments of the BR corresponding to different sinuses (Fig. 2A and B).

### Measured fibrous and muscular parts of the basal ring

**Figure 3:**
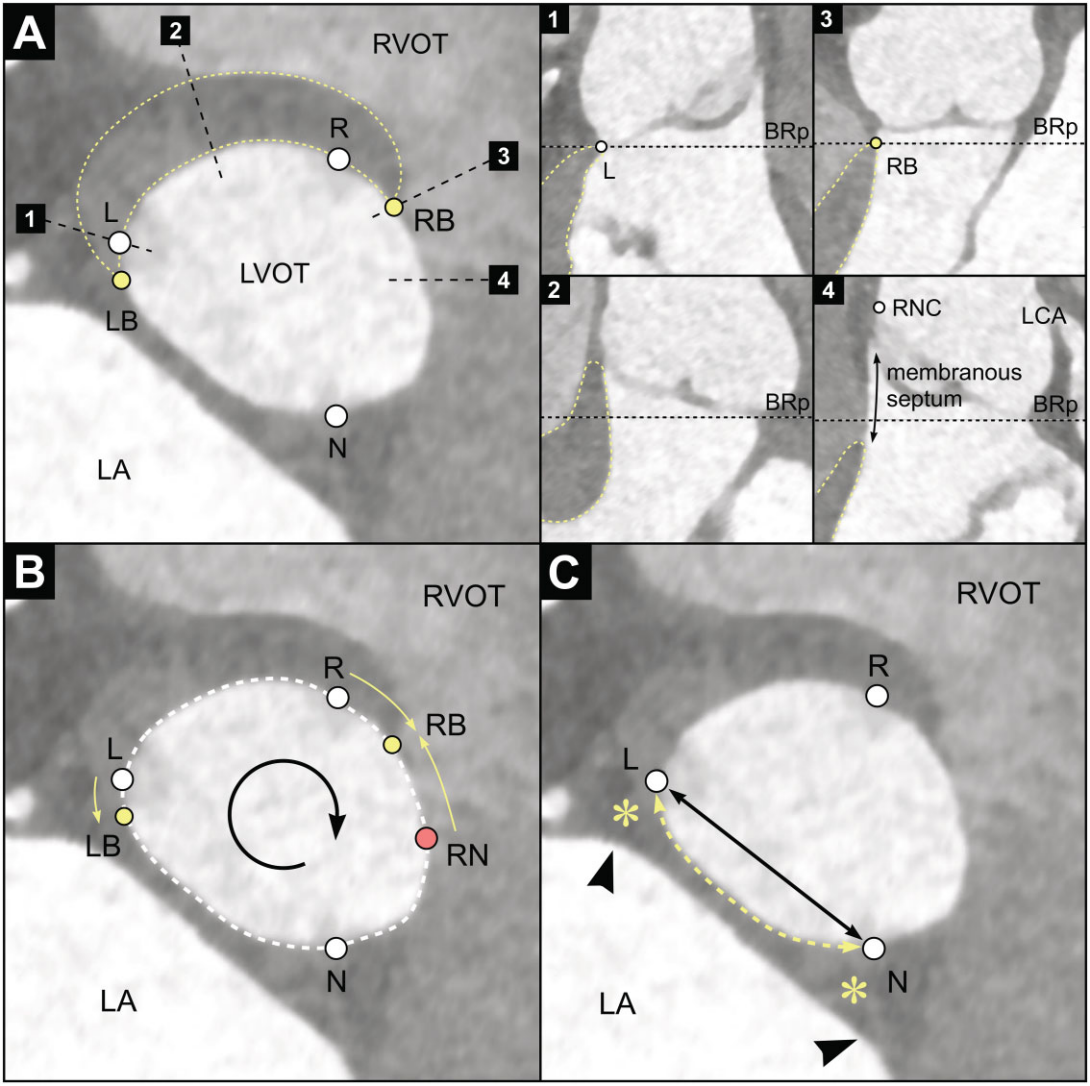
Computed tomography angiography of the aortic root at the level of the basal ring. (**A**) White points labelled L, R and N mark the nadirs of the left, right and non-coronary sinuses, respectively. The yellow dotted line encircles the left ventricular muscle. Yellow points labelled LB and RB mark the left and right border of the muscular part of basal ring, respectively. Black dotted lines with numbers show locations of perpendicular vertical transections through the basal ring plane, which are shown on the right as sub-Figs 1–4. In the sub-figures, the black dotted line shows the location of the basal ring plane (BRp). (**B**) Measured distances along the basal ring perimeter. We measured distances between the left (LB) and right (RB) border of the muscular part of BR and the nadirs of the left (L) and right (R) sinuses and the projection of the right-non commissure to the basal ring (RN). Distances are positive in the clockwise direction viewed from the aortic root and negative in the counterclockwise direction. In this patient, the L–LB distance is –1.7 mm, R–RB distance 6.5 mm and RNC-RB distance –8.7 mm. (**C**) Estimates of intertrigonal distance. Intertrigonal distance was estimated by direct distance between left (L) and non-coronary (N) nadir (black line with arrows) and distance along BR perimeter between left and non-coronary nadir (yellow dashed line with arrows). Asterisks mark the left and right fibrous trigones. Arrowheads mark external landmarks for the trigones seen as indentations when viewed from the LA. LA: left atrium; LCA: left coronary artery; LVOT: left ventricular outflow tract; RNC: right-non commissure; RVOT: right ventricular outflow tract.

The borders of the muscular part of the BR were marked on all CTAs with adequate visibility and then the lengths of the segments of the BR corresponding to muscular and fibrous part were calculated. These segments were termed measured muscular and measured fibrous part of the BR. Based on these 2 measurements, we calculated the fractions of measured muscular and fibrous BR, by dividing each measurement by BR perimeter. We also calculated the distances along the BR perimeter between the left border and nadir of the left sinus and between the right border and RNC projection to the BR and nadir of the right sinus (Fig. 3B).

### Estimated muscular and fibrous parts of the basal ring

As an alternative to direct measurements, which were not possible in all patients, we split the BR into 2 segments—1 that is mainly fibrous and 1 that is mainly muscular. These segments were termed estimated muscular and estimated fibrous BR. The estimated fibrous BR included the non-coronary sinus segment of BR and part of left coronary sinus segment of BR from the nadir of the left sinus to the projection of the left-non commissure to BR. The remaining BR defined the estimated muscular BR (Fig. 2C). We also calculated fractions of estimated muscular and fibrous BR, by dividing each measurement by BR perimeter.

### Estimated intertrigonal distances

The distance between right and left fibrous trigone was previously estimated in cadaver and 3D echocardiography studies based on the surface landmarks on the mitral annulus as seen from the left atrium [8, 9]. Anatomic studies have shown that the left fibrous trigone is located at the nadir of the left sinus and the right fibrous trigone at the nadir of the non-coronary sinus [10]. In our study, we used 2 distances to estimate the intertrigonal distance: direct distance between the nadirs of the left and non-coronary sinuses and distance along the BR perimeter between the same 2 points (Fig. 3C).

### Statistical methods

Statistical data analysis was performed using JASP v 0.14.1 (University of Amsterdam, Netherlands). Normal distribution of variables was assessed using the Shapiro–Wilk test. Continuous variables are reported as mean and standard deviation if normally distributed and as median and interquartile range otherwise. Categorical variables are reported as numbers and percentages. Differences in continuous variables were analysed using Student’s *t*-test, Mann–Whitney *U*-test and Friedman test with Conover’s *post hoc* comparisons and Bonferroni’s correction. Differences in categorical variables were analysed using the chi-squared test. A *P*-value of <0.05 was considered statistically significant.

Propensity score matching was used to select patients with comparable age, body size and sex using the available demographic data (age, weight, height, body surface area and sex). Nearest neighbour method of matching was used with ratio of 1:1 and calliper size was set to 0.2 SD of logit of propensity score (MatchIt algorithm, R Foundation for Statistical Computing, Vienna, Austria). Differences in matched variables were analysed using paired samples *t*-test and Wilcoxon signed-rank test for continuous, and McNemar’s chi-squared test for categorical data.

## RESULTS

**Figure 4:**
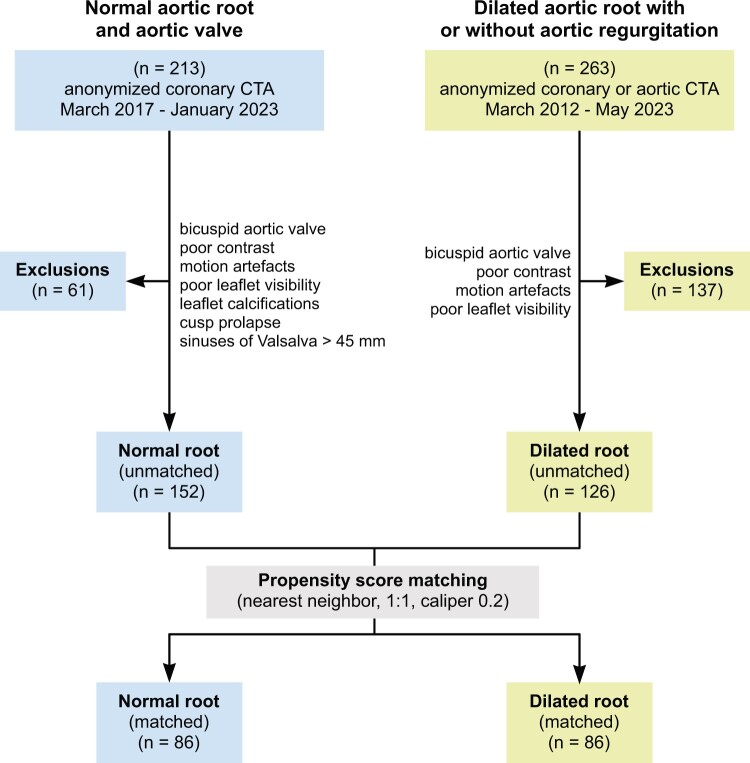
Study population selection. CTA: computed tomography angiography.

For the normal root group, we collected 213 CTAs, 61 were excluded from further analysis due to 1 or more exclusion criteria. In the remaining 152 CTAs, the aortic valves were tricuspid, without calcifications, with no visible coaptation defects and without root dilatation. Based on the morphology of leaflets and absence of calcification and coaptation defects, we assumed all analysed aortic valves functioned normally. For the dilated root group, we collected 263 CTAs, 137 were excluded from further analysis due to 1 or more exclusion criteria, leaving 126 CTAs for detailed analysis (Fig. 4). Details of CTA studies are presented in Supplementary Material.

The age distribution was similar in both groups with a median of 57 years; however, the patients with dilated root were predominantly male (84%) and had higher height, weight and body surface area (Table 1). After propensity score matching, we obtained 86 patients in each group (Supplementary Material Fig. 1A). The matched patients were comparable for all variables, with standardized mean difference of <0.1 (Supplementary Material Fig. 1B).

**Table 1:** Patient characteristics before and after propensity score matching

	All	Normal root (unmatched)	Dilated root (unmatched)	SMD	Normal root (matched)	Dilated root (matched)	SMD
*n*	278	152	126		86	86	
Age (years), median (IQR)	57 (20)	56 (19)	58 (20)	–0.026	56 (15)	57 (20)	–0.024
Height (cm), mean (SD)	176 (11)	172 (9)	181 (10)	0.851	177 (8)	178 (10)	0.068
Weight (kg), mean (SD)	83 (17)	78 (15)	88 (17)	0.648	84 (14)	84 (15)	0.003
BSA (m^2^), mean (SD)	2.00 (0.25)	1.92 (0.23)	2.10 (0.23)	0.775	2.03 (0.20)	2.03 (0.21)	0.013
Male sex, *n* (%)	190 (68)	84 (55)	106 (84)	0.790	67 (78)	68 (79)	0.032

BSA: body surface area; IQR: interquartile range; SD: standard deviation; SMD: standardized mean difference.

The sinuses of Valsalva diameter, commissural diameter and BR diameter were all significantly larger in dilated roots (Table 2). The BR diameter was on average 4 mm and 2 mm larger in unmatched and matched dilated roots, respectively. Unmatched dilated roots had a slightly more circular BR as evidenced by 3% lower ratio between maximal and minimal BR diameter; however, in matched patients, this difference was not observed.

**Table 2:** Basal ring segmental measurements

	All	Normal root (unmatched)	Dilated root (unmatched)	*P*-value	Normal root (matched)	Dilated root (matched)	*P*-value
*N*	278	152	126		86	86	
Sinuses of Valsalva diameter (mm), median (IQR)	38.3 (15.3)	33.2 (5.4)	49.3 (7.7)	<0.001^a^	35.3 (4.9)	48.5 (7.7)	<0.001^b^
Commissural diameter (mm), median (IQR)	31.1 (15.0)	27.8 (4.2)	43.2 (8.8)	<0.001^a^	29.0 (3.4)	42.3 (8.3)	<0.001^c^
BR diameter (mm), median (IQR)	26.2 (4.4)	24.7 (3.9)	28.3 (4.2)	<0.001^a^	25.8 (2.9)	28.0 (4.7)	<0.001^c^
Max/min BR diameter ratio^d^, median (IQR)	1.30 (0.12)	1.32 (0.10)	1.28 (0.13)	0.027^a^	1.29 (0.10)	1.30 (0.15)	0.394^b^
BR segments corresponding to the 3 sinuses							
Right BR segment (mm), median (IQR)	26.4 (5.5)	25.0 (4.5)	28.9 (5.2)	<0.001^a^	26.4 (3.5)	28.8 (5.9)	<0.001^c^
Left BR segment (mm), median (IQR)	27.2 (5.3)	26.3 (5.1)	28.7 (6.1)	<0.001^a^	27.3 (4.8)	28.6 (6.9)	<0.001^c^
Non BR segment (mm), median (IQR)	28.1 (6.0)	26.0 (4.3)	30.8 (5.5)	<0.001^a^	27.1 (3.8)	30.7 (4.9)	<0.001^b^
Fraction of right BR (%), median (IQR)	32.4 (2.7)	32.3 (2.4)	32.4 (3.1)	0.782^a^	32.3 (2.1)	32.4 (3.0)	0.283^b^
Fraction of left BR (%), median (IQR)	33.5 (3.2)	33.8 (3.1)	33.0 (3.1)	0.003^e^	33.7 (3.1)	33.2 (3.3)	0.094^b^
Fraction of non BR (%), median (IQR)	34.4 (4.3)	34.0 (4.6)	34.6 (3.7)	0.026^a^	33.9 (4.6)	34.6 (3.3)	0.082^b^
Estimated muscular and fibrous BR							
Estimated muscular BR (mm), median (IQR)	40.0 (8.5)	38.2 (7.2)	42.2 (8.6)	<0.001^a^	39.7 (5.3)	42.0 (10.0)	<0.001^c^
Estimated fibrous BR (mm), median (IQR)	42.2 (8.5)	38.9 (6.0)	46.2 (7.5)	<0.001^a^	40.7 (4.9)	45.5 (6.9)	<0.001^b^
Fraction of est. muscular BR (%), mean (SD)	48.4 (3.7)	49.1 (3.4)	47.6 (3.8)	<0.001^e^	49.2 (3.5)	47.9 (3.7)	0.029^b^
Fraction of est. fibrous BR (%), mean (SD)	51.6 (3.7)	50.9 (3.4)	52.4 (3.8)	<0.001^e^	50.1 (3.5)	52.1 (3.7)	0.029^b^
Estimates of the intertrigonal distance							
N to L distance along BR perimeter (mm), median (IQR)	27.3 (5.7)	25.2 (4.4)	30.3 (5.3)	<0.001^a^	26.3 (3.6)	30.1 (5.0)	<0.001^c^
Direct N to L distance (mm), median (IQR)	24.5 (4.8)	22.5 (4.1)	26.5 (4.2)	<0.001^a^	23.3 (3.5)	26.3 (3.6)	<0.001^b^
Fraction of N to L distance (%), median (IQR)	33.3 (4.0)	32.9 (3.5)	34.0 (4.5)	<0.001^a^	32.8 (3.1)	34.1 (4.2)	<0.001^b^

a Mann–Whitney *U*-test.

b Paired samples *t*-test.

c Wilcoxon signed-rank test.

d ratio between maximal and minimal BR diameter.

e Student’s *t*-test.

BR: basal ring; IQR: interquartile range; L: nadir of the left sinus; N: nadir of the non-coronary sinus.

Segments of the of the BR corresponding to the 3 sinuses were all significantly longer in dilated roots. The fraction of the BR occupied by the right aortic sinus was not significantly different between the 2 groups; however, the fraction of the left sinus was smaller and the fraction of the non-coronary sinus larger in unmatched dilated roots. Similar differences were observed in matched roots, although not statistically significant.

In the dilated root group, 19 (15.1%) patients had grade 1, 23 (18.3%) grade 2, 47 (37.3%) grade 3 and 20 (15.9%) grade 4 aortic regurgitation. The jet of aortic regurgitation was central in 87 patients with functional aortic regurgitation and eccentric in 22 patients with cusp prolapse. Right cusp prolapse was observed in 13 patients, left cusp prolapse in 7 patients, non-coronary cusp prolapse in 1 patient and combined right and left cusp prolapse in 1 patient. The fraction of the right BR segment was not different between dilated roots with and without right cusp prolapse [median 33.2% (interquartile range 3.0%, *n* = 14) vs 32.4% (interquartile range 3.1%, *n* = 111), *P* = 0.346, Mann–Whitney *U*-test].

The dimensions of estimated muscular and fibrous BR were significantly larger in dilated roots. The fraction of estimated fibrous BR was significantly larger in dilated roots, although the difference was small at ∼1–2% in both matched and unmatched groups.

The estimated intertrigonal distances and the fraction of the BR perimeter occupied by the intertrigonal segment were all significantly larger in dilated roots. The difference in fraction was ∼1% and was observed in both matched and unmatched groups.

The border between the fibrous and the muscular part of the BR was not clearly visible on all CTAs, particularly the border beneath the right sinus. The main determinant of visibility was the amount of contrast in the right atrium and right ventricle, which marked the border of the membranous septum in the BR plane (Fig. 3A). The border beneath the left sinus was visible in almost all CTAs. Because of poor visibility, 57 CTAs (20.5%, 26 normal CTAs and 31 dilated root CTAs) were excluded from analysis of measured muscular and fibrous BR and only complete cases were used. Comparison between complete cases and cases with missing values is presented in the Supplementary Material Table S1.

Both measured muscular and measured fibrous BR were longer in patients with dilated roots; however, the fractions of measured fibrous and muscular BR were not significantly different between the 2 groups (Table 3). The left border of the muscular BR was located at the nadir of the left sinus, with a mean distance between left border and left nadir of <1 mm in both groups. The right border was located on the right segment of the BR, on average 8 mm from the nadir of the right sinus and 5 mm from the projection of the RNC to the BR.

**Table 3:** Measured muscular and fibrous part of basal ring in all patients with adequate CTA (*n* = 221)

	All	Normal root (unmatched)	Dilated root (unmatched)	*P*-value	Normal root (matched)	Dilated root (matched)	*P*-value
*n*	221	126	95		60	60	
Measured muscular BR (mm), median (IQR)	34.5 (8.0)	33.1 (7.0)	36.8 (8.1)	<0.001^a^	34.4 (7.7)	35.8 (8.7)	0.005^b^
Measured fibrous BR (mm), median (IQR)	46.6 (9.0)	44.1 (6.2)	52.2 (9.1)	<0.001^a^	45.6 (4.6)	51.2 (10.0)	<0.001^b^
Fraction of meas. muscular BR (%), median (IQR)	42.1 (5.5)	42.4 (4.6)	41.5 (5.9)	0.074^a^	42.2 (4.3)	42.1 (6.3)	0.262^b^
Fraction of meas. fibrous BR (%), median (IQR)	57.9 (5.4)	57.6 (4.6)	58.5 (5.9)	0.073^a^	57.8 (4.3)	57.9 (6.3)	0.262^b^
Distance from L nadir to LB (mm), median (IQR)	–0.3 (3.0)	–0.4 (2.5)	–0.1 (3.5)	0.645^a^	–0.2 (2.6)	–0.5 (3.8)	0.722^c^
Distance from RN to RB (mm), median (IQR)	–5.2 (3.4)	–5.0 (2.9)	–5.6 (4.4)	0.398^a^	–4.9 (2.6)	–5.3 (4.1)	0.877^b^
Distance from R nadir to RB (mm), median (IQR)	8.3 (3.6)	7.9 (3.4)	9.3 (4.6)	<0.001^a^	8.3 (3.3)	9.5 (3.9)	0.058^c^

When 1 or both patients in the matched pair had inadequate computed tomography angiography, they were both excluded from further analysis. Hence only 60 patients in each matched group.

a Mann–Whitney *U*-test.

b Wilcoxon signed-rank test.

c Paired samples *t*-test.

BR: basal ring; IQR: interquartile range; L: left; LB: left border of the muscular part of BR; R: right; RB: right border of muscular part of BR; RN: right-non commissure projection to the BR.

The 3 cusp insertion lengths in dilated aortic roots were significantly larger than the corresponding lengths in normal roots (*P*-values for all pairs were <0.001 using Mann–Whitney *U*-test). In normal roots, the right and non-coronary cusp insertion lengths were of equal size and longer than the left one (Table 4), whereas in dilated roots, the non-coronary cusp insertion length was the longest, the left the shortest and the right cusp insertion length in between. These differences were observed in matched and unmatched groups.

**Table 4:** Cusp insertion lengths in normal and dilated roots

	All	Normal root (unmatched)	Dilated root (unmatched)	Normal root (matched)	Dilated root (matched)
*n*	278	152	126	86	86
Mean cusp insertion length (mm), median (IQR)	57.0 (17.5)	51.7 (8.4)	69.4 (13.2)	54.1 (6.6)	67.7 (12.9)
Right cusp insertion length (mm), median (IQR)	57.7 (16.4)	52.4 (9.0)	69.0 (12.2)	54.9 (5.9)	67.4 (9.4)
Left cusp insertion length (mm), median (IQR)	55.5 (15.8)	50.3 (7.9)	66.0 (11.5)	53.2 (6.3)	64.7 (11.7)
Non-coronary cusp insertion length (mm), median (IQR)	58.6 (21.1)	52.3 (8.8)	73.7 (13.8)	54.5 (6.9)	71.7 (13.0)
Friedman test (right versus left versus non-coronary cusp insertion length)	<0.001	<0.001	<0.001	<0.001	<0.001
Right versus left^a^ (*P*-value)	<0.001	<0.001	<0.001	<0.001	<0.001
Left versus non-coronary^a^ (*P*-value)	<0.001	<0.001	<0.001	<0.001	<0.001
Right versus non-coronary^a^ (*P*-value)	0.006	0.309	<0.001	0.912	<0.001

a Conover’s *post hoc* comparisons with Bonferroni correction. All cups insertion lengths in dilated roots were significantly longer than in normal roots in both matched and unmatched groups.

IQR: interquartile range.

## DISCUSSION

BR enlargement is common in patients with dilated aortic root and functional aortic regurgitation in tricuspid aortic valves [11–13]; however, the segmental dilatation has not been thoroughly studied before. Our study has confirmed the association of aortic root aneurysm and BR dilatation. The difference in BR diameter between the 2 unmatched groups was on average 3.6 mm, which corresponds to an 11 mm (15%) increase in BR perimeter. However, all segments of the BR did not dilate equally. Dilatation was largest in the non-coronary segment of the BR and smallest in the left segment of the BR. The difference in the fraction between the normal and dilated roots for the non-coronary segment was small in the range of 1%. The fraction of the right segment of the BR did not change significantly even in the presence of right cusp prolapse.

The non-coronary segment of the BR is the major component of the fibrous part of BR, hence a small enlargement of ∼1–2% in the proportion of the estimated fibrous part of the BR was also noted in patients with dilated aortic root. However, the border between muscular and fibrous part was set arbitrarily for this measurement. This difference was not seen in the measured fraction of the fibrous BR, where measurement was possible in 79.5% of patients, although a similar trend was observed in the unmatched groups. Neumann *et al.* have shown similar results [14]. They used CTAs to show that muscular portion of the BR was reduced in patients with aortic valve regurgitation compared to normal adults and patients with aortic valve stenosis (37.5% vs 40.5% and 44.3%). The drawbacks of their study were: a small number of patients with aortic regurgitation (*n* = 25), measurements were performed in mid-systole as opposed to end diastole and data regarding the root size in aortic regurgitation patients were not reported.

Root dilatation is primarily a disease of the aortic wall and as the root dilates, it also stretches the BR. In our study, the dilatation of sinuses of Valsalva and the commissural diameter was much more pronounced than BR dilatation. Conversely, aortic root remodelling procedure has been shown to reduce BR to a certain extent solely by replacing and reducing the sinuses even without additional annuloplasty [15]. In our study, the dilatation in the non-coronary part of the root was not limited to the BR but was also evident in cusp insertion lengths, which are part of the aortic sinuses. In normal aortic roots, the right and non-coronary cusp insertion lengths were identical and slightly longer than the left cusp insertion length; however, in dilated roots, the non-coronary cusp insertion was the longest, followed by the right, whereas the left was the shortest. Cusp insertions define the border of sinuses of Valsalva, and an increase in cusp insertion length means that the corresponding sinus has increased either horizontally, vertically, or both.

The intertrigonal distance as well as the fraction of BR corresponding to intertrigonal distance increased in dilated roots. However, in degenerative mitral regurgitation, the intertrigonal distance (the fibrous part of the mitral annulus) does not increase despite annular dilatation in the muscular part of mitral annulus [8, 9]. Additionally, the mitral annulus size correlates well with tricuspid annulus size but not with aortic annulus size [8]. Intertrigonal distance is directly related to the fibrous part of the BR; however, it was not measured directly in our study, because of difficulty of determining the edge or the centre of either of the trigones on CTA as they merge with aorto-mitral curtain (Fig. 3C). In both cadaver and echocardiographic studies, the position of the 2 fibrous trigones was defined by approximate external landmarks in the left atrium, which were not validated with anatomical or histological studies [8, 9]. It seems that intertrigonal distance is more closely related to the aortic valve than to the mitral valve.

Additionally, our data have shown that the nadir of the left sinus was an excellent marker for the left border of the muscular part of BR with mean distance of <1 mm in both normal and dilated roots. The right border, however, was more variable and usually located between the nadir of the right sinus and RNC projection to BR, closer to the latter.

### Limitations

The main limitation of the study was the inability to determine the borders of the muscular part of BR in some patients due to the lack of contrast agent in the right atrium and the right ventricle. The study only applies to tricuspid aortic valves. Due to the study design, echocardiographic confirmation of normal aortic valve function was not possible in the normal root group. More CTAs were excluded in the dilated root group due to 2 main reasons. The 1st is a higher proportion of bicuspid valves, which is expected in the group of patients who had aortic valve-sparing surgery. The 2nd is a higher proportion of CTAs with poor cups visibility, because some of the CTAs were not ECG gated. Additional limitations of CTAs are reported in the Supplementary Material.

## CONCLUSION

To our knowledge, this study is the 1st to show that BR dilatation is present in all segments in patients with dilated aortic roots with subtle differences between the 3 sinuses. These findings support the need to stabilize and reduce the entire BR using internal or external ring or suture [16–19] and not only a part of it [3] when performing aortic valve repair in dilated root patients. Unlike in the mitral valve, aortic annular dilatation includes both muscular and fibrous parts, with a tendency to more dilatation in the intertrigonal area and non-coronary segment of the BR.

## Supplementary Material

ivae029_Supplementary_Data

## Data Availability

The data underlying this article will be shared on reasonable request to the corresponding author.

## Notes

Presented at the 37th EACTS meeting, Vienna, Austria, October 2023.

## ABBREVIATIONS

BR: Basal ring
CTA: Computed tomography angiography
RNC: Right-non commissure
