# Supplementary material for: Segmental analysis of aortic basal ring dimensions in normal and dilated tricuspid aortic roots
Source: Interdiscip Cardiovasc Thorac Surg. 2024 Feb 28;38(3):ivae029. doi: 10.1093/icvts/ivae029 (PMC10927323; doi:10.1093/icvts/ivae029)
Supplement: ivae029_Supplementary_Data [file ivae029_supplementary_data.zip › Supplemental material.docx]

**
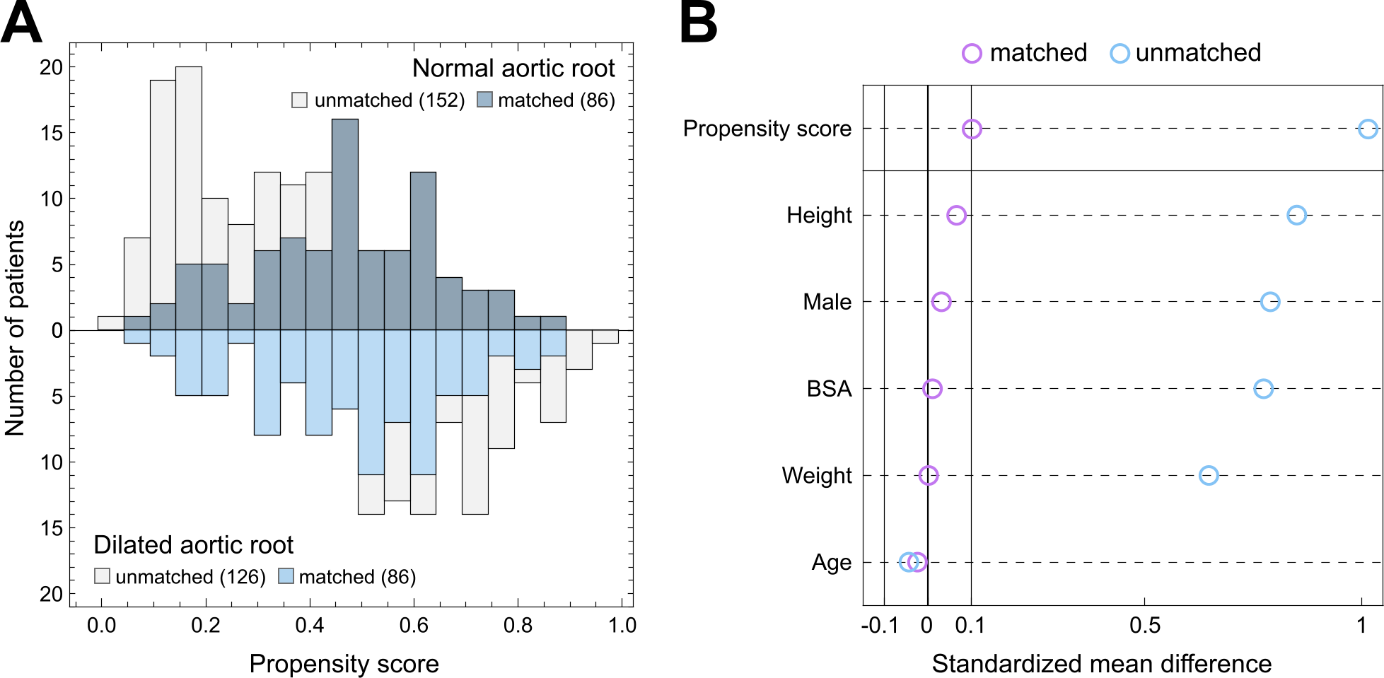
**

**Supplemental figure 1.** Propensity score matching. **(A)** Mirrored histogram showing the propensity score distribution in unmatched (light grey) and matched (blue) samples in the normal aortic root (top) and dilated aortic root group (bottom). **(B)** Love plot showing standardized mean difference before and after matching for the selected variables and propensity score. BSA = body surface area.

**CTA additional data**

One of the additional limitations of the study was the heterogeneity of the CTAs in terms of the year of the study, study protocol (aortic, thoracic or coronary), and CT device used, which is unavoidable in a multi-centric, retrospective study of a relatively rare pathology.

CTAs were performed between 2012 and 2023. Between 2012 and 2015 we had 4 patients (1.4%), in 2017 – 12 patients (4.3%), 2018 - 24 patients (8.6%) and all the remaining 238 patients (85.6%) had CTA in the last 5 years. Median and IQR for year of CTA was 2022 (IQR 1) for the normal group and 2021 (IQR 3) for the pathology group.

The CTA were performed in diastolic phase (R-R interval at mean of 72.0% ± 5.7%). All CTAs in the normal group had slice thickness 0.6 mm, whereas in the dilated root group 70 (55.5%) had slice thickness of 0.6 mm, 10 (7.9%) 0.75 mm, 3 (2.4%) 0.9 mm and 41 (32%) 1 mm, 2 patients had missing metadata on slice thickness. Pixel spacing in the normal group was 0.385 (IQR 0.052) vs 0.424 (IQR 0.096) in the dilated root group, p < 0.001 (Mann-Whitney U-test).

All CTAs in the normal and most of the CTAs in the dilated root group were performed on Siemens (Berlin, Germany) devices (SOMATOM Force (181), SOMATOM Drive (58), SOMATOM Definition AS (16) and Biograph mCT (18)), Additionally there were 4 CTAs in the dilated root group performed on the Phillips (Amsterdam, Netherlands) Ingenuity CT and 1 on the GE HealthCare (Chicago, IL, USA) Revolution EVO.

**Supplemental table 1.** Comparison between patients with adequate and inadequate CTAs for direct measurement of muscular and fibrous parts of basal ring.

|  | Adequate CTA | Inadequate CTA | P-value |
| --- | --- | --- | --- |
| n | 221 | 57 |  |
| Height (cm), mean (SD) | 176 (11) | 176 (9) | 0.773^1^ |
| Weight (kg), mean (SD) | 82 (16) | 84 (18) | 0.450^1^ |
| Body surface area (m^2^), mean (SD) | 2.00 (0.24) | 2.01 (0.26) | 0.611^1^ |
| Age (years), median (IQR) | 56 (20) | 58 (15) | 0.029^2^ |
| Male gender, n (%) | 154 (69.7%) | 36 (63%) | 0.123^3^ |
| Aortic regurgitation, n (%) | 81 (36.6%) | 28 (49%) | 0.086^3^ |
| Normal root size, n (%) | 126 (57%) | 26 (45%) | 0.123^3^ |

^1^Student’s t-test, ^2^Mann-Whitney U test, ^2^chi-squared test
